# Design and Fabrication of a Low-Voltage OPAMP Based on a-IGZO Thin-Film Transistors

**DOI:** 10.3390/nano16020084

**Published:** 2026-01-08

**Authors:** Arturo Torres-Sánchez, Isai S. Hernandez-Luna, Francisco J. Hernández-Cuevas, Cuauhtémoc León-Puertos, Norberto Hernández-Como

**Affiliations:** Centro de Nanociencias y Micro y Nanotecnologías, Instituto Politécnico Nacional, Mexico City 07700, Mexico; atorressa@ipn.mx (A.T.-S.); ishernandezl@ipn.mx (I.S.H.-L.); cleon@ipn.mx (C.L.-P.)

**Keywords:** a-IGZO, thin-film transistors, operational amplifiers, bandwidth, phase margin

## Abstract

In the last few years, Thin Film Transistors (TFTs) based on materials such as amorphous Indium–Gallium–Zinc Oxide (a-IGZO) have gained interest in large-area and low-cost electronics due to their high carrier mobility, high on/off current ratio, low off-state current, and steep subthreshold slope. These characteristics make IGZO TFTs suitable for radio-frequency identification (RFID) tags, analog-to-digital converters (ADCs), logic circuits, sensors, and analog components, including operational amplifiers (OPAMPs). This work presents the implementation and characterization of an OPAMP based on n-type a-IGZO TFTs fabricated on glass substrate. Two previously reported design strategies were integrated: a positive feedback network to increase the output impedance and a topology to enhance the transconductance of the driver transistors, both in the differential input stage. A gain of 26 dB, a bandwidth of 2.4 kHz, a gain–bandwidth product (GBWP) of 48 kHz, and a phase margin of 64° were obtained, which confirms the reliability of the design and the fabrication process.

## 1. Introduction

In the last few years, Thin-Film Transistors (TFTs) based on metal oxides, such as Indium–Gallium–Zinc Oxide (IGZO), have gained interest due to their properties such as high field-effect mobility [1,2,3,4,5,6,7,8,9,10,11,12,13], the possibility of being fabricated by low or even room temperature processes [3,4,9,10,11,12,13,14], as well as their excellent electrical stability [4]. Also, recent research has emphasized that IGZO remains a key semiconductor for flexible, large-area, and low-power electronics due to its uniformity and low leakage current [15]. These characteristics, combined with a low production cost [2,4,5,7,9,11,16], make them an attractive alternative in large-area and low-cost electronics. Because of this, a-IGZO TFTs have been implemented in a wide range of circuits and systems, including radio frequency identification (RFID) tags [1,4,8,16], analog-to-digital converters (ADCs) [4], logic circuits and sensors [3], display drivers [1,3,8,16], ring oscillators [4,8], and analog components including operational amplifiers (OPAMPs) [4].

However, unlike conventional CMOS technology, OPAMPs implemented with IGZO TFTs present inherent limitations in mobility and transconductance-to-current ratio, which limits the achievable gain in basic configurations. Therefore, it is necessary to employ design topologies that increase the effective gain of the circuit, namely, the use of positive feedback networks, optimized current mirrors, multiple amplification stages, or pseudo-CMOS [1]. These strategies make it possible to compensate for the limitations of individual devices, improving key parameters such as the gain-to-bandwidth product.

The gain of an amplifier is determined by the product of the transconductance (gm) of the driver transistor and the load resistance R_L_. Moreover, metal oxide TFTs lack p-type devices, which avoids the implementation of active current sources as loads, a common topology for achieving high output impedance in amplifiers [1]. Due to this limitation, additional techniques such as positive feedback are essential to achieve high gain amplifiers [1,16].

In this work, an OPAMP based on a-IGZO n-type TFTs operating in incremental mode was implemented. Two previously reported topologies were used to achieve a gain-to-bandwidth ratio appropriate to the characteristics of our devices. The first topology incorporates a positive feedback network, which allows an increase in the circuit’s output impedance, while the second is based on increasing the transconductance of the driver transistor, both applied in the OPAMP’s differential input stage.

## 2. Fabrication Process

**Figure 1:**
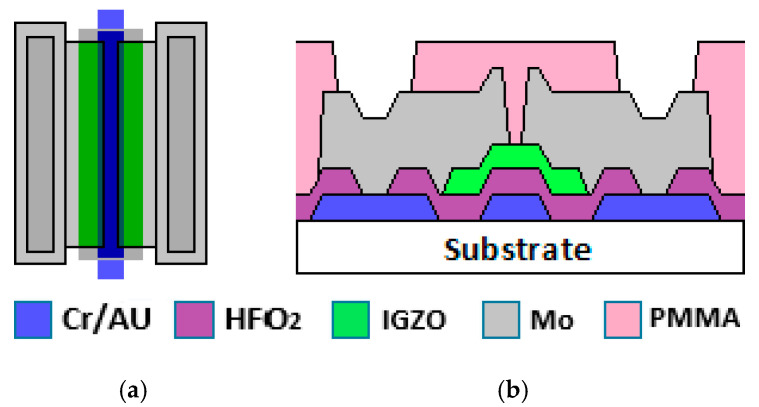
(**a**) Top view and (**b**) cross-sectional schematic of an a-IGZO TFT used as the basic cell in OPAMP design.

The OPAMP is based on n-type TFTs, which were designed using a 5 µm fabrication process. Figure 1a,b show the top and cross-sectional views, respectively, of the bottom gate structure of an a-IGZO TFT, which forms the basic cell of the designed OPAMP. The fabrication process begins with the cleaning of the Corning Eagle XG glass substrate with acetone and isopropyl alcohol. Afterwards, Cr/Au is sequentially deposited by electron beam evaporation, and the TFT gate electrode is formed by lithography and wet etching. As the gate dielectric, Hafnium Oxide (HfO_2_) was deposited by the atomic layer deposition (ALD) technique at a process temperature of 150 °C. To have contact with the Cr/Au metal, the dielectric layer was defined with 6:1 BOE (Buffered Oxide Etchand) wet etching. Subsequently, an a-IGZO semiconductor film was deposited at room temperature by RF-sputtering using a Kurt J. Lesker target; this layer was defined with H_2_O:HCl (4:1) wet etching. To activate the carriers in the a-IGZO film, a heat treatment at 150 °C (10 °C steps) in air for one hour was realized. As metallic source and drain contacts, a Molybdenum (Mo) film was obtained by sputtering, also at room temperature, while the contact definition was performed by a lift-off process. Finally, poly(methyl methacrylate) (PMMA) was coated as a passivation layer by spin-coating, followed by a heat treatment consisting of applying a thermal ramp from 100° to 200 °C, with increments of 10 °C/min, during 20 min. To make contact with the Mo pads, the PMMA was etched with reactive ion etching (RIE).

The fabrication process consisted of six mask layouts designed using open-access available CAD tools. The lithography was performed using Heidelberg direct-write equipment. Profile measurements yielded a-IGZO film and dielectric thicknesses of 10 nm and 26 nm, respectively, while the HfO_2_ dielectric constant of 11.1 was obtained for the metal/insulator/metal structures.

**Figure 2:**
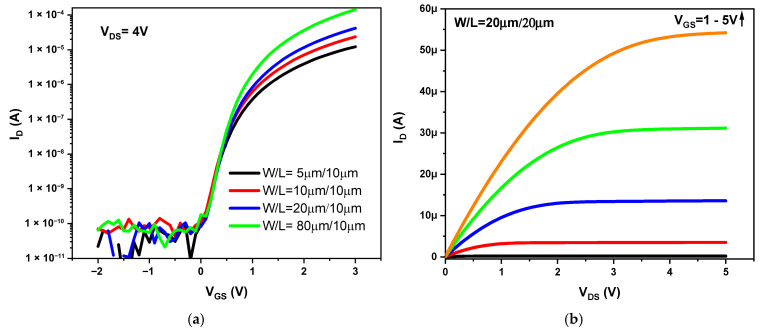
(**a**) Transfer characteristic in saturation region for a-IGZO TFTs with different channel widths, and (**b**) output curve for an a-IGZO TFT with (W/L = 20 μm/20 μm).

In Figure 2a the measured transfer characteristic graph in the saturation region is presented for TFTs with different width dimensions (W = 5, 10, 20, and 80 µm) and length (L = 10 µm) of channel, while in Figure 2b the output graph of a TFT with W/L = 20 µm/20 µm is shown. The field-effect mobility (µ_fet_) in the saturation regime was calculated using the equation $\mu_{fet}=(2\cdot L\cdot m)/(W\cdot Cox)$, where m is the slope of the $\sqrt{I_{D}}\;\;vs.\;V_{GS}$ plot, W and L are the channel width and length, respectively, and Cox is the capacitance of the gate dielectric. The threshold voltage (Vth) was extracted by extrapolating the linear region of the $\sqrt{I_{D}}\;vs.\;V_{GS}$ curve until it intersects the *x*-axis. The I_ON_/I_OFF_ ratio and subthreshold slope (SS) were extracted from the (log I_D_) graph in the saturation regime. A mobility of up to 24 cm^2^/Vs was obtained for the device with largest W, whereas the mobility decreased as the channel width was increased. This may be due to the fact that the V_th_ increased as the TFT channel width increased. The rise in value of V_th_ with a larger W may be due to the fact that as the channel width increases, more current flows through it, thus increasing the temperature in the a-IGZO material due to the Joule effect. It has been measured [17] and simulated [18] that self-generated heat is mainly confined to the center of the channel. As the channel width increases, it becomes more difficult for the heat to dissipate to the adjacent materials in the direction of W. Therefore, heat dissipation will be lower in TFTs with higher W, leading to a more pronounced charge trapping effect [19] and thus increasing V_th_. Table 1 summarizes the parameters extracted from the TFT devices such as mobility, V_th_, I_ON_/I_OFF_, and SS.

## 3. OPAMP Design and Simulation

**Figure 3:**
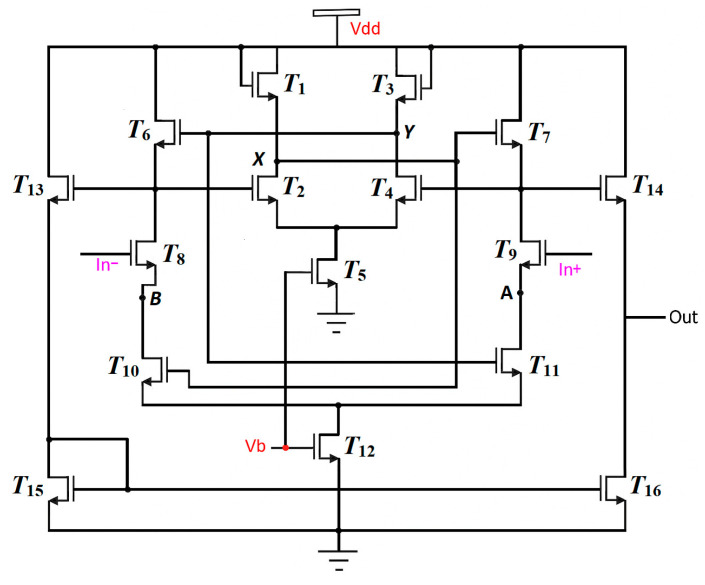
Schematic circuit of the designed OPAMP based on n-type a-IGZO TFTs.

Figure 3 presents the schematic circuit of the designed operational amplifier, composed exclusively of n-type TFTs. The first stage corresponds to a differential pair implemented by transistors T6–T9. This is complemented by a proposed topology to increase transconductance, consisting of transistors T10 and T11, whose gates are connected to the positive feedback network integrated by T1–T4. Transistors T5 and T6 act as bias current sources for the positive feedback network and for the differential stage, respectively. The bias voltage V_b_ was adjusted so that all devices operated in the saturation region. Finally, the second stage, composed of transistors T13–T16, implements a current mirror whose function is to transform the differential signal into a single-ended output.

### Circuit Analysis

**Figure 4:**
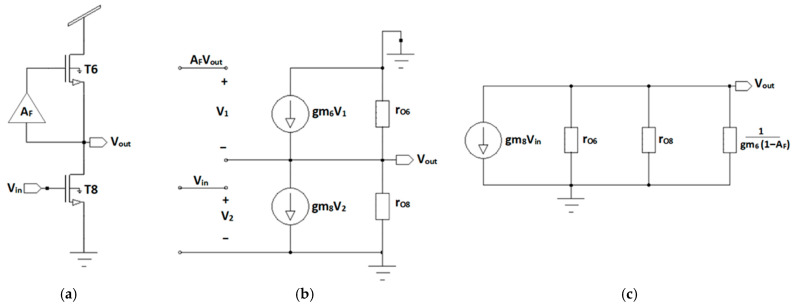
(**a**) Common-source amplifier with positive feedback, (**b**) its corresponding small-signal model, and (**c**) the equivalent circuit.

In order to analyze the first stage, the half circuit formed by transistors T6 and T8 is examined, together with a block labeled $A_{F}$, as is presented in Figure 4a. This circuit consists of a common-source amplifier, in which transistor T6 acts as the load and T8 as the driver device, while block A_F_ corresponds to a positive feedback circuit, which has been previously used to increase the output impedance in this configuration.

Figure 4b shows the corresponding small-signal model. As can be seen, the control voltage ($V_{1}$) of the controlled current source is determined by the difference between the output voltage scaled by the feedback factor ($A_{F}$) and the output voltage itself, that is,

(1) $$V_{1}=V_{out}(A_{F}-1)$$

On the other hand, the control voltage of the controlled current source of the driver transistor (T8) is directly the input voltage, V_2_ = V_in_. Since a controlled current source, whose value depends on the voltage between its terminals, can be modeled by a resistor [20], and also considering the direction of the current, the equivalent circuit shown in Figure 4c is obtained. As can be seen, the resulting circuit is formed by three resistors in parallel together with the current source. Applying Ohm’s law, the expression for the small-signal voltage gain ($A_{V}$) of this stage is obtained:

(2) $$A_{V}=\frac{V_{out}}{V_{in}}=-g_{m8}\left[r_{o8}∥r_{o6}∥\frac{1}{g_{m6}\left(1-A_{F}\right)}\right]$$

where $r_{o8}$ and $r_{o6}$ are the output resistances of T8 and T6, while $g_{m8}$ and $g_{m6}$ are their respective transconductances. On the other hand, the positive feedback circuit consists of two cascaded stages of common-source inverting amplifiers with saturated load. This configuration means that the output has the same phase as the input signal. In this case the feedback configuration used consists only of one inverting amplifier circuit (T3 and T4) since the input to it comes from the output of the circuit formed by T7 and T9 belonging to the opposite input (In +). Therefore, the small-signal gain of the positive feedback circuit can be expressed as the following [20]:

(3) $$A_{F}\approx \frac{g_{m4}}{g_{m3}}=\frac{\sqrt{{\left(W/L\right)}_{4}}}{\sqrt{{\left(W/L\right)}_{3}}}$$

In addition, one way to enhance the circuit gain is to increase the transconductance of the driver transistors (T8, T9) by placing elements in their respective sources that behave as “negative resistors”, so that, when the current increases due to a rise in the amplitude of V_in_, its drain voltage decreases, increasing V_GS_ and, therefore, the transconductance of the driver. One practical way to implement this negative resistance is placing transistors connected in a differential configuration. In [1], the authors connected the transistors using a common-source stage with source degeneration, where the gate of T10 was connected to the source of driver T9 (node A) and the gate of T11 to the source of driver T8 (node B). However, this configuration has the disadvantage that, for example, when the amplitude of the Vin+ input increases, due to the opposite V_in-_ input node operating under differential mode, the node B decreases its amplitude and therefore, the gate of T10 receives a lower voltage that reduces its bias and, therefore, decreases the current of that branch. As a result, the feedback does not reinforce the rising driver current and, consequently, does not improve the common-mode rejection of the circuit.

To address this drawback, in this work we propose an alternative topology for enhanced transconductance of the driver transistor. The circuit still maintains the common-source configuration with source degeneration; however, instead of connecting the gates of the transistors to opposite nodes (A or B), they are connected to the drains of the positive feedback network (nodes X or Y). For instance, when the amplitude of Vin+ increases, the positive feedback network formed by T1 and T2 generates a signal in phase with Vin+ at node X. This signal, applied to the gate of T10, increases the current in that branch, which causes a decrease in the potential at node B and, consequently, an increase in the differential mode of the amplifier. Without considering effects such as channel modulation and channel effect, and taking into account that T10 acts as an active load, the equivalent transconductance of this circuit is given by the following [20]:

(4) $$G_{m}=\frac{g_{m8}}{1-\frac{g_{m8}}{g_{m10}}}$$

where $g_{m8}$ and $g_{m10}$ represent the transconductances of transistors T8 and T10, respectively. In this common-source configuration with source degeneration, the quadratic dependence of the current on the overdrive voltage is attenuated [16], which improves the linearity of the stage and, consequently, achieves more stable and predictable circuit behavior.

Finally, the approximate small-signal voltage gain of the OPAMP is the product of the voltage gains in each stage:

(5) $$A_{V,Total}=A_{V1}\cdot A_{V2}$$

where the gain of the first stage, considering $A_{F}$ ≈ 1 and replacing Equation (4) by g_m8_ in Equation (2), is expressed as

(6) $$A_{V1}=-\frac{g_{m8}}{1-\frac{g_{m8}}{g_{m10}}}\left(r_{o1}∥r_{o2}\right)$$

while the gain of the differential-to-single-ended stage is given by [21]

(7) $$A_{V2}=\frac{g_{m14}r_{o16}}{2\left(1+g_{m14}r_{o16}\right)}\left(1+\frac{g_{m13}g_{m16}}{g_{m14}\left(g_{m13}+g_{m15}\right)}\right)$$

where $\;g_{m13}$, $g_{m14}$, $g_{m15}$, and $g_{m16}$ correspond to the transconductances of transistors T13, T14, T15, and T16, respectively, while $r_{o16}$ represents the output resistance of T16. Since in this current mirror, the current flowing through T13 and T15 is replicated by T14 and T16, and also considering that all transistors have the same dimensions of W/L, their transconductances are equal. Under this condition, Equation (7) can be expressed in a simplified form:

(8) $$A_{V2}\approx \frac{3}{4}\frac{g_{m14}r_{o16}}{\left(1+g_{m14}r_{o16}\right)}$$

With gm_14_ r_o16_ ≫ 1, the second-stage gain is close to 0.75. Therefore, the approximate total gain of the OPAMP is given by

(9) $$A_{V,Total}=-\frac{3}{4}\frac{g_{m8}}{\left(1-\frac{g_{m8}}{g_{m10}}\right)}\left(r_{o1}∥r_{o2}\right)$$

The overall gain of the OPAMP can be increased by making the transconductance of $g_{m8}$ larger than that of $g_{m10}$. This can be achieved by increasing the dimensions of transistor T8 relative to T10 or by adjusting the bias voltage V_b_. Table 2 summarizes the dimensions of each TFT making up the OPAMP.

**Figure 5:**
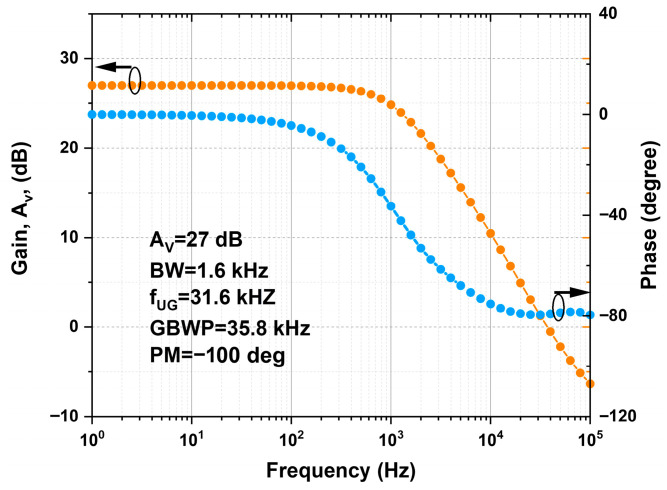
Simulated frequency response of the designed OPAMP. Markers highlight the curve associated with each axis.

The OPAMP design was verified and optimized using T-SPICE software v16.0, using the a-Si (Level 15) model. The transistor dimensions were adjusted to achieve good gain while maintaining a sufficient phase margin. Figure 5 shows the simulation of the frequency response of the proposed OPAMP. The results obtained were as follows: open loop gain of 27 dB, bandwidth (BW) of 1.6 kHz, unity gain frequency (f_UG_) of 31.6 kHz, gain bandwidth product (GBWP), and a phase margin (PM) of −100°. The simulations were carried out with bias voltages V_DD_ = 5.1 V and V_b_ = 1.6. The AC differential input signals (Vin+ and Vin−) were applied superimposed on an offset voltage of 2.9 V.

## 4. Experimental Results

**Figure 6:**
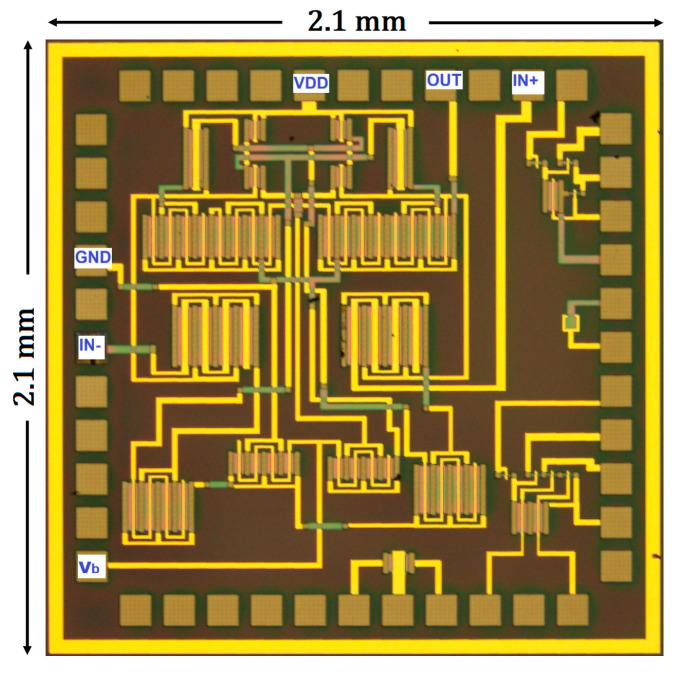
Micrograph (not at scale) of the fabricated OPAMP on glass substrates.

Figure 6 shows the micrograph of the proposed OPAMP, fabricated on a glass substrate and comprising exclusively n-type IGZO TFTs in enhance mode. To minimize the Vth variation associated with channel width, multi-finger TFTs were employed in the layout design.

**Figure 7:**
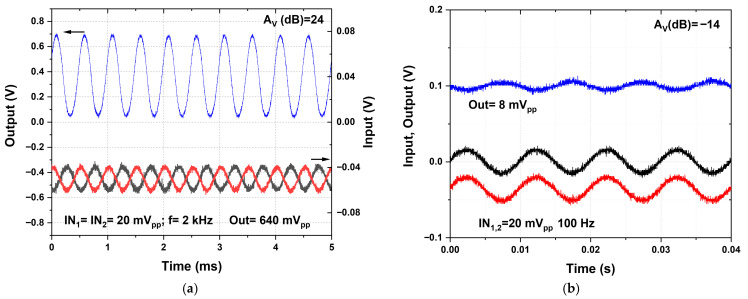
Measured transient response of the fabricated OPAMP in (**a**) differential input mode and (**b**) common input mode.

Figure 7a presents the time-domain response of the fabricated OPAMP. Experimental characterization of the OPAMP was carried out using an oscilloscope (Tektronix MSO3034, Beaverton, OR, USA), a function generator (RIGOL DG822Pro, Suzhou, China), and a semiconductor parameter analyzer (Keithley 4200A-SCS, Clevaland, OH, USA). All measurements were performed under dark and ambient conditions. The supply voltages of V_DD_ = 5.5 V and V_b_ = 1.8 V were supplied by an external voltage source; two input sinusoidal signals were applied in differential mode, both at a frequency of 2 kHz, with an amplitude of 20 mV_pp_ and an offset voltage of 2.5 V. The measured output signal shows a differential mode gain defined as

(10) $$\;A_{V}=20\;log\left|\frac{V_{out}}{V_{IN1}-V_{IN2}}\right|$$

An experimental gain of 24 dB and a phase shift of −47° were measured. Furthermore, it is observed that the output signal is clear and well-defined, confirming that the OPAMP operates correctly in differential mode, thus validating the proposed design as well as the reliability of the fabrication process.

On the other hand, Figure 7b shows the common-mode gain. In this case, two input signals were applied in phase, both of 20 mVpp at a frequency of 100 Hz, obtaining an output signal with a gain of −14 dB. The common mode rejection ratio (CMRR) was calculated as

(11) $$CMRR=20\;log\left(\frac{A_{D}}{A_{CM}}\right)$$

where A_D_ and A_CM_ are the differential-mode and common-mode gains, respectively. The calculated values yield a CMRR of 40 dB at low frequencies (100 Hz). A higher CMRR directly translates into better noise suppression, thereby improving the output signal and allowing more accurate measurement of small-amplitude signals. In the case of circuits fabricated with IGZO TFTs, optimizing the CMRR is key to ensuring proper performance, for instance, in bio-sensing applications, where noise robustness is critical.

**Figure 8:**
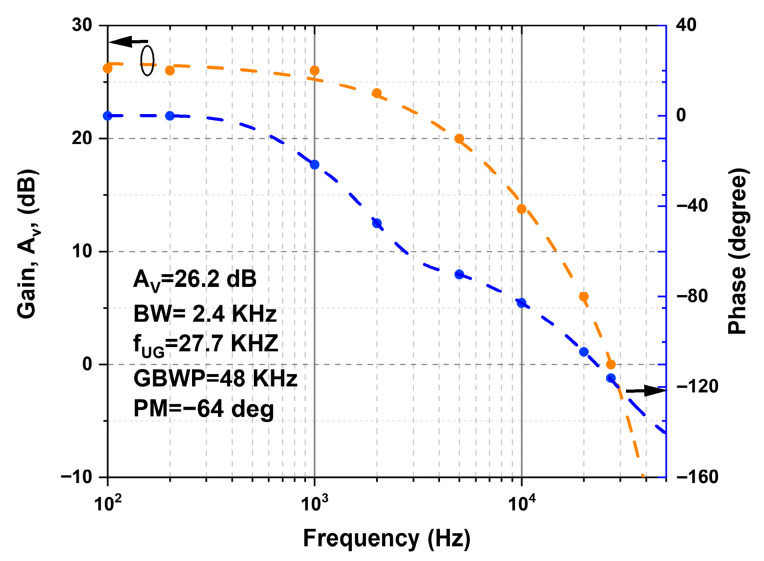
Frequency response of the fabricated OPAMP. The arrows highlight the curve associated with each axis.

Figure 8 shows the frequency response of the fabricated OPAMP. The low-frequency gain was 26 dB, with a BW of 2.4 kHz, a GBWP of approximately 48 kHz, and a f_UG_ frequency of 27.7 kHz, while a PM of 64° was obtained, indicating that the amplifier response remains far from the critical condition in which the circuit would tend to oscillate. When compared with the simulation results, differences are observed in the BW, f_UG_, and PM values. These differences are mainly attributed to the fabrication process of the TFTs that make up the OPAMP, as well as to the influence of device parameters (threshold voltages, parasitic capacitances, contact resistances, etc.) that are not taken into account or modeled with total accuracy in the simulation. However, the general trend of the frequency response remains consistent, validating the proposed design.

Table 3 compares the performance of the proposed OPAMP with previous works reported in the literature. In terms of bias voltage, the designs of Ref. [16] and Ref. [22] require 20 V and 15 V, respectively, while the proposed OPAMP works with 5.5 V, which represents an advantage for low-power applications. Regarding the open-loop gain (Av), the value of 26 dB obtained in this work is lower than 50 dB [16] and 32.7 dB [4], but higher than 19 dB [3] and 19.6 dB [23], placing it in an intermediate performance range characteristic of IGZO-based OPAMPs.

Regarding the BW and GBWP, the reports of Ref. [16] and Ref. [23] exhibit high values (15.2 kHz and 4806 kHz in [16]; 350 kHz and 3342 kHz in [23]), associated both with the higher bias voltage and with topology differences. The OPAMP developed in this work shows a BW of 2.4 kHz and a GBWP of 48 kHz, closer to those of (9.3 kHz; 279.9 kHz) [22] and (25 kHz; 223 kHz) [3]. The calculated BW is noticeably lower than those reported in [5,16]. This behavior is primarily attributed to the OPAMP operating at a lower supply voltage (V_DD_ = 5.5 V); a lower value of V_DD_ decreases the available overdrive voltage in the differential input stage, therefore lowering the drain current and the effective gm of the driver devices. Considering that the unity-gain bandwidth is proportional to gm and capacitance, the combination of moderate gm, parasitic capacitances from interconnects and measurement setup, and the reduced supply voltage results in a lower achievable bandwidth. Further improvement of the OPAMP bandwidth can be pursued through several strategies, such as increasing the effective transconductance of the input pair using wider devices, employing higher-κ gate dielectrics to increase gate capacitance, and reducing parasitic overlap and routing capacitances at the layout level. An additional and promising approach is the integration of negative-capacitance (NC) structures into oxide-based TFTs [24]. Finally, the PM of −64° achieved is higher than [16] (−24°), [16] (−21.5°), [4] (35.8°) and closer to [3] (−70°). The PM remains sufficiently far from the critical condition of −180°, thereby providing stable operation in low-frequency applications.

## 5. Conclusions

An OPAMP based on n-type a-IGZO TFTs was implemented and characterized. The integration of a positive feedback network allowed an increase in the output impedance, while increasing the transconductance in the differential stage improved the overall gain of the circuit without compromising its stability. Experimental results showed a gain of 24 dB at 2 kHz and a phase margin of 64°, confirming the dynamic stability of the design. The OPAMP operated with a supply voltage of 5.5 V, which is considerably lower than that reported in most previous works based on a-IGZO TFTs, demonstrating better energy efficiency and improved compatibility with low-power electronic systems. Furthermore, the obtained CMRR of 40 dB demonstrates good common-mode suppression, an essential aspect for noise-critical applications such as biosensing.

## Figures and Tables

**Table 1:** Extracted electrical parameters of the fabricated a-IGZO TFTs.

W/L (µm/µm)	V_th_ (V)	µ_fet_ (cm^2^/Vs)	I_Dmax_ (µA)	I_ON_/I_OFF_	SS (mV/Decade)
W = 5; L = 10	0.67	24.0	12.2	2.1 × 10^5^	177
W = 10; L = 10	0.73	23.4	23.6	2.5 × 10^5^	167
W = 20; L = 10	0.79	21.3	41.7	5.6 × 10^5^	137
W = 80; L = 10	0.89	19.2	143.5	1.5 × 10^6^	127

**Table 2:** Channel dimensions (W/L) of the TFTs used in the proposed OPAMP.

	W/L (µm/µm)		W/L (µm/µm)		W/L (µm/µm)
T1, T3	67/5	T8, T9	800/20	T15, T16	560/5
T2, T4	71/5	T10, T11	120/15		
T5	280/5	T12	560/5		
T6, T7	200/20	T13–T116	560/5		

**Table 3:** Comparison of previously designed OPAMPs based on metal-oxide TFTs.

Circuit	[16]	[22]	[3]	[4]	[23]	This Work
Year	2022	2018	2015	2023	2019	2025
Technology	Metal-Oxide	Metal-Oxide	a-IGZO	a-IGZO	a-IGZO	a-IGZO
Topology	Positive-feedback	Positive-feedback	Positive-feedback	Positive-feedback	N-MOS diode	Positive-feedback
V_DD_	20	15	6	15	10	5.5
A_V_ (dB)	50	29.5	19	32.67	19.6	26
BW (kHz)	15.2	9.33	25	15	350	2.4
GBWP (kHz)	4806	279.9	223	-	3342	48
*f*_UG_ (kHz)	500	180.2	330	200	1600	27.7
PM	−24	−21.5	−70	−35.8	−102	−64

## Data Availability

The original contributions presented in this study are included in the article. Further inquiries can be directed to the corresponding author.
